# EXPLORING THE ASSOCIATIONS BETWEEN DAILY SLEEP AND THE PITTSBURGH SLEEP QUALITY INDEX (PSQI) AMONG OLDER ADULTS

**DOI:** 10.1093/geroni/igae098.3521

**Published:** 2024-12-31

**Authors:** Hye Won Chai, Alyssa Gamaldo, Charlene Gamaldo, Abigail Stephan, Christine Phillips, Lesley Ross

**Affiliations:** Clemson University, Clemson, South Carolina, United States; Clemson University, Clemson, South Carolina, United States; Johns Hopkins Howard County Medical Center, Columbia, Maryland, United States; Clemson University, Clemson, South Carolina, United States; Clemson University Institute for Engaged Aging, Seneca, South Carolina, United States; Clemson University, Clemson, South Carolina, United States

## Abstract

While the Pittsburgh Sleep Quality Index (PSQI) is widely used in sleep research, it is based on adults’ retrospective memory which may be subject to recall bias. Daily measures of sleep using smartphones may supplement PSQI by providing a more ecologically valid snapshot of respondents’ sleep patterns. However, there is a limited understanding about whether and how daily reports of sleep (over 14 days) map onto PSQI scores among older adults. This study aims to explore the associations between diverse measures of daily sleep and PSQI in later life. We used baseline survey and diary data from the Everyday Function Intervention Trial on 95 older adults (age range: 60-88). Daily measures of sleep included overall means and day-to-day variations in sleep quality, difficulty falling asleep, difficulty staying asleep, and feeling refreshed after wake across two weeks. Results revealed that higher overall mean levels of difficulty falling asleep was associated with higher total PSQI score (i.e., worse sleep). Higher overall mean levels of sleep quality were associated with several PSQI subscales (less sleep disturbance, better sleep efficiency, and higher subjective quality of sleep). Higher day-to-day variations in sleep quality was associated with better PSQI sleep efficiency and higher variations in feeling refreshed after wake was associated with longer PSQI sleep latency. These results suggest that daily measures of sleep may be tapping onto different dimensions of PSQI and further highlight the need to incorporate diverse measures of sleep to have a more holistic understanding of sleep patterns in later life.

